# Image Guidance in Ablation for Hepatocellular Carcinoma: Contrast-Enhanced Ultrasound and Fusion Imaging

**DOI:** 10.3389/fonc.2021.593636

**Published:** 2021-03-05

**Authors:** Yasunori Minami, Masatoshi Kudo

**Affiliations:** Department of Gastroenterology and Hepatology, Faculty of Medicine, Kindai University, Osaka, Japan

**Keywords:** ablation therapy, contrast-enhanced ultrasound, fusion imaging, hepatocellular carcinoma, poor conspicuity, precise ablation

## Abstract

The ultrasound (US) imaging technology, including contrast-enhanced US (CEUS) and fusion imaging, has experienced radical improvement, and advancement in technology thus overcoming the problem of poor conspicuous hepatocellular carcinoma (HCC). On CEUS, the presence or absence of enhancement distinguishes the viable portion from the ablative necrotic portion. Using volume data of computed tomography (CT) or magnetic resonance imaging (MRI), fusion imaging enhances the three-dimensional relationship between the liver vasculature and HCC. Therefore, CT/MR-US fusion imaging provides synchronous images of CT/MRI with real-time US, and US-US fusion imaging provides synchronous US images before and after ablation. Moreover, US-US overlay fusion can visualize the ablative margin because it focuses the tumor image onto the ablation zone. Consequently, CEUS and fusion imaging are helpful to identify HCC with little conspicuity, and with more confidence, we can perform ablation therapy. CEUS/fusion imaging guidance has improved the clinical effectiveness of ablation therapy in patients with poor conspicuous HCCs. Therefore; this manuscript reviews the status of CEUS/fusion imaging guidance in ablation therapy of poor conspicuous HCC.

## Introduction

Ablation therapy is a minimally invasive treatment option, and percutaneous ultrasound (US)-guided ablative treatments, including radiofrequency ablation (RFA), and microwave ablation (MWA), have successfully managed hepatocellular carcinoma (HCC) (1–6). However, patients with difficult conditions for ablation therapy require multiple treatment sessions due to the limitation of US guidance. Poorly conspicuous HCC is not easily targeted on B-mode US guidance and accounts for 5.2–38.8% of planning US for ablation therapy (7–10). The success of percutaneous ablation therapies primarily depends on correct targeting through an imaging technique and the suitable placement of the needle electrode into the target tumor thereby optimizing local tumor control.

The US imaging technology has experienced radical improvement, and advances in hardware and software have helped to overcome the problem of poor conspicuity on US. Presently, contrast-enhanced ultrasound (CEUS) is widely used in clinical practice and it provides significant contribution to the diagnosis of HCC (11, 12). On CEUS, we can distinguish the viable portion of HCC from the ablative necrotic one by the presence or absence of enhancement, and we can perform an image-guided ablation of this viable HCC. In addition, other technological advancements allow two-dimensional (2D) multiplanar reconstruction (MPR) images of CT or MRI to display in the same plane as US images. Consequently, fusion imaging becomes a powerful technique to detect poor conspicuous HCC on US. Moreover, image fusion technology contributes to the progress of ablation therapy as well as other fields.

This article reviews the principles, clinical applications, and techniques of US image-guidance in ablation therapy including CEUS and fusion imaging.

## CEUS-Guided Ablation

### Contrast Agents and Pharmacokinetics

Contrast agents, such as SonoVue/Lumason (Bracco, Milan, Italy) and Sonazoid (GE Healthcare, Waukesha, WI, USA), are microbubbles containing a low-solubility gas enveloped by a phospholipid shell. These microbubbles provide stable non-linear oscillation in a low-power acoustic field because of their hard shells, thereby displaying vascular pattern in real-time. However, Kupffer cells of the liver engulf the contrast agent (especially sonazoid). Therefore, sonazoid microbubbles can accumulate in the liver parenchyma, thereby displaying enhancement of the liver parenchyma for a considerable period (13).

Regarding CEUS, the standard protocol for the examination of the liver consists of two main phases: the vascular and Kupffer phases (14–17). The vascular phase can be divided into three phases. These include the arterial phase (15 s after injection and lasting for 25–30 s), portal phase (30 s after injection and lasting for 2–3 min), and late vascular phase (4–7 min). The Kupffer phase starts 10 min post-injection of Sonazoid. The key diagnostic feature of HCC with SonoVue/Lumason is the hyper-enhancement seen in the arterial phase followed by a clearance seen in the portal and/or late phase. Similarly, we observe a hyper-enhancement in the arterial phase followed by defect in the Kupffer phase with sonazoid. In addition, repeated contrast injections are also useful for diagnosis of HCC. This procedure termed “defect reperfusion imaging” or “the re-injection technique” can diagnose HCC in the presence of arterial enhancement(s) in a defective lesion/wash-out (18–20).

### Technique of CEUS Guidance

Prior to ablation therapy, we can use CEUS to assess the HCC lesion size, number, margins, and relationship with the surrounding liver vasculature. The diagnostic accuracy of CEUS (using SonoVue or Sonazoid) for poorly conspicuous HCC is 93.8–100%, similar to contrast-enhanced CT or MRI (21–24). Thus, CEUS facilitates needle placement in HCC poorly conspicuous on B-mode US, such that the defect/wash-out lesion signifies the target insertion point. Moreover, we can administer US contrast agents repeatedly in order to guide percutaneous ablation of multiple lesions.

Sometimes, it is difficult to differentiate local HCC tumor progression from ablative necrotic areas because both similarly show hypoenhancement lesions in the late vascular/Kupffer phase. Consequently, the defect reperfusion imaging becomes very useful in the confirmation of viable HCCs that are otherwise undetectable on US (20). Nevertheless, it is sometimes difficult to depict HCC with low-contrast images in the late vascular/Kupffer phase in severe liver cirrhosis. In other words, we experience a weak contrast brightness intensity of liver parenchyma due to a decrease of portal blood flow and number of Kupffer cells in the liver. Therefore, in such situations, we must administer a higher dose of US contrast agent to patients in order to improve contrast brightness intensity.

### Evidence of CEUS-Guided Ablation Therapy

CEUS guidance in ablation therapy has increased operators’ confidence and improved the outcome. It was reported that the success rate at first session of CEUS was significantly higher than that in B-mode US guidance for poor conspicuous HCC (94.7 *versus* 65.0%, p = 0.043) (25). Some previous cohort studies found that the number of sessions was significantly smaller with CEUS guidance than with B-mode US guidance (26, 27). Another study demonstrated that the local control rate was higher with CEUS guidance than with B-mode US guidance (85.3 *versus* 66.4% at 2 years, p = 0.044) (28). Moreover, CEUS easily recognizes serious acute complications including active bleeding or hepatic infarction, which is not the case with B-mode US guidance (29). CEUS may show active hemorrhage as extravasation of microbubbles through the needle tract and hepatic infarction as a hypoenhancement lesion.

## Fusion Imaging-Guided Ablation

### Applications of Fusion Imaging

The commercial image fusion platforms include Real-Time Virtual Sonography (RVS) (Hitachi, Tokyo, Japan), volume navigation (v-nav) (GE Healthcare, Waukesha, WI, USA), SmartFusion (Canon Medical systems, Tokyo, Japan), eSie Fusion Imaging (Siemens Healthcare, Erlangen, Germany), and PercuNav (Philips, Andover, MA, USA).

**Figure 1:**
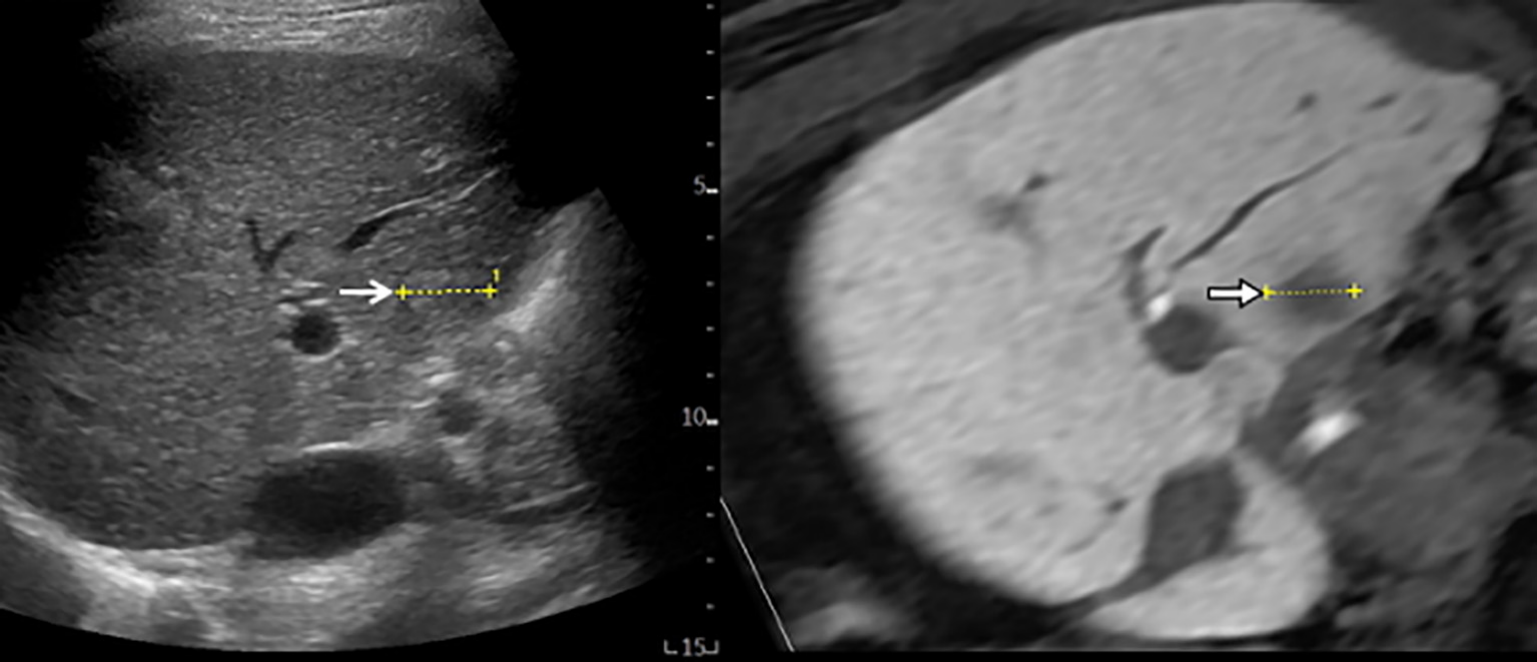
MR-US fusion imaging. MRI and US images with HCC measuring 1.8 cm in diameter are well-matched. B-mode US (left) shows a slightly hyperechoic nodule with ill-defined HCC (open arrow) from intercostal view. Hepatobiliary phase image of gadoxetic acid-enhanced MRI (right) shows low signal intensity with ill-defined HCC (arrow).

**Figure 2:**
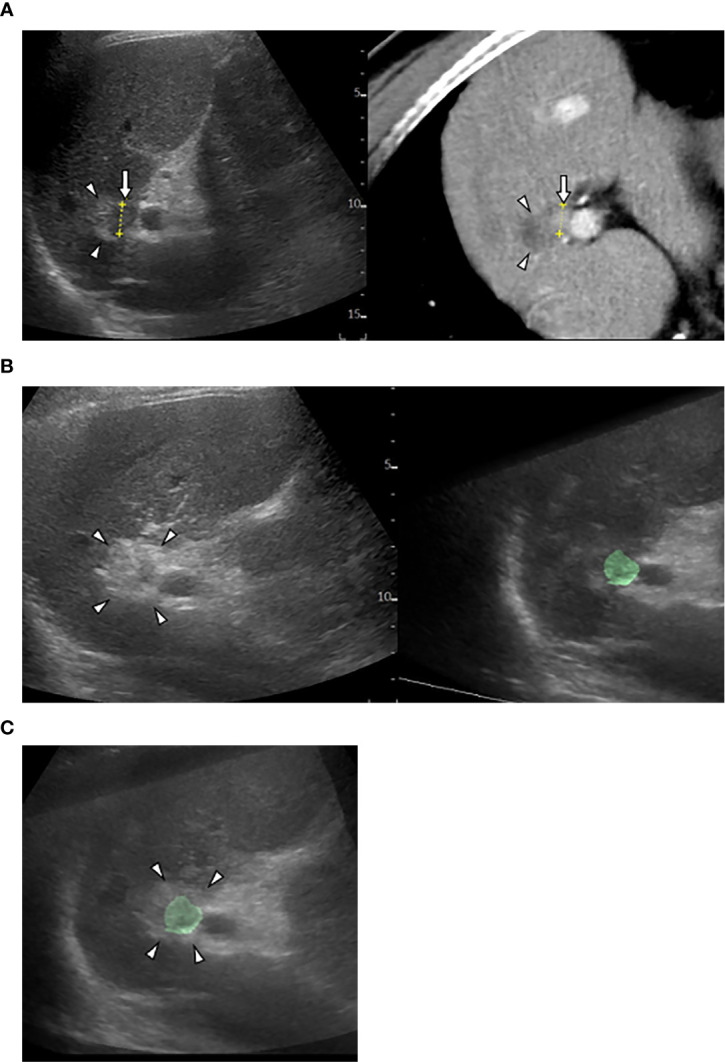
US-US fusion imaging and US-US overlay fusion. **(A)** CT-US fused image shows locally progressed hepatocellular carcinoma (HCC) (arrows) touching ablative necrosis (arrow heads) due to previous ablation. **(B)** US-US fusion imaging displays HCC colorized as green before ablation (right) and ablative hyperechoic zone (arrowheads) due to the present ablaion (left). **(C)** US-US overlay fusion demonstrates the green colorized HCC inside the ablative hyperechoic zone concentrically.

Cross-sectional MPR images from 3D-volume data allow virtual sonographic images, and magnetic tracking based on mapping of a 3D magnetic field. When using fusion imaging, we can obtain spatial information from the relationship between the magnetic field generator and the magnetic sensor attached to the US probe. By integrating the spatial information between the US probe and 3D volume data, a 2D-MPR image can show the same plane and move synchronously with real-time US images (30–32). CT/MR-US fusion imaging provides synchronous images of CT/MRI with real-time US (**Figure 1**). US-US fusion imaging provides synchronous US images before and after ablation (**Figure 2**). Moreover, it can visualize the ablative margins on US before ablation because it projects the tumor image onto the ablation zone.

### Technique of Fusion Imaging Guidance

To operate effectively using fusion imaging technology, there is a need to match (co-register) the 3D image datasets with the real-time US; that is, we need to register the reference points near the tumor carefully. When the CT/MRI and US images are well-matched, inconspicuous HCC can be identified. Thus, CT/MR-US fusion imaging can increase the detectability of small HCCs compared to B-mode US. However, the gap in co-registration can persist in some situations. For example, imaging gap occurs because the depths of breath-holds in CT and sonographic examinations vary. Originally, CT/MR-US fusion imaging offer no support for synchronized action of breathing with any diagnostic US scanners at present. However, the priority is for the operator to catch the tumor location on US because the operator inserts the therapeutic needle watching on an US monitor under patients’ breathing. Not so strictly image matching is necessary for inserting the therapeutic needle into the tumor in fact.

In contrast, succinct co-registration accuracy (in mm) can be required when assessing an ablative margin. For high quality images, a 3D-UD volume has to be obtained by a swing scanning with slow and steady speed. For high-quality image matching, we need to register the reference points near the tumor before and after the ablation more carefully.

### Evidence of Fusion Imaging-Guided Ablation Therapy

#### CT/MR-US Fusion Imaging

According to some retrospective studies, the success and local tumor progression rates using RFA guided by CT/MR-US fusion imaging (for poorly conspicuous HCC on B-mode US) were 94.4–100% and 0–8.3%, respectively (33–37). According to a prospective study by Ahn et al. (38), CT/MR-US fusion imaging significantly improved the tumor visibility and operators’ confidence compared to B-mode US alone (p < 0.001). Consequently, the recurrence-free survival rates were 86.0 and 75.8% at 12- and 24-months, respectively. The cumulative incidences of local tumor progression were 3.2 and 4.7% at 12- and 24-months, respectively.

#### US-US Fusion Imaging and US-US Overlay Fusion

Successful ablation therapy requires a wide ablation zone (including the tumor with ablative safety margin) in order to restrain local tumor progression. Although fusion imaging improves the visualization of HCC, some factors limit a 5-mm safety margin. These include large tumor size, tumor morphology, vasculature around the tumor, subcapsular tumor location, and gas bubbles in ablation zone (39, 40). Gas formation in particular, could envelope the tumor leading to a blind assessment of the ablative margin on US. Therefore, this ablative margin assessment technique was revised in order to overcome this challenging issue of a 5-mm safety margin.

US-US fusion imaging is used to compare images before and after ablation in a side-by-side manner, and US-US overlay fusion visualizes the ablative margin by focusing on the ablation zone of the projected tumor image (41–45). We achieved 5-mm safety margins in 89.3% (108/121) of HCC nodules using the US-US overlay fusion technique compared to 47.0% (213/453) in the conventional guidance group (P < 0.01). Two-year local tumor progression rates were 0.8% (1/121) with US-US overlay fusion and 6.0% (27/453) with conventional guidance (P = 0.022) (46).

## The Combination Guidance of CEUS and Fusion Imaging

**Figure 3:**
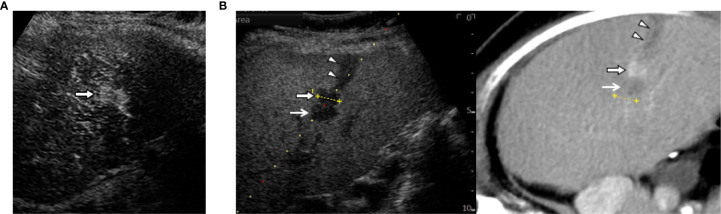
The combination of contrast-enhanced US (CEUS) and fusion imaging. **(A)** CEUS with Sonazoid shows arterial enhancement of locally progressed hepatocellular carcinoma **(HCC)** (arrow). **(B)** The combination of Kupffer image on contrast-enhanced US (CEUS) and fused CT image demonstrates viable HCC (arrow) sandwiched between an ablated tract (arrow heads) and a necrotic tumor (open arrows).

Operators may attempt CEUS fused with CT/MR image when CT/MR-US fusion imaging fails to identify HCCs. Either CEUS or fusion imaging provides an inadequately favorable condition for ablation therapy. Therefore, the combination of fusion imaging and CEUS is the last option (**Figure 3**). Even in difficult situations, we observed no significant differences in the number of treatment sessions required to obtain technical success of ablation between CEUS, fusion imaging, and the combination guidance (47). In addition, the combined guidance could be preferred for recurrent subcentimeter HCCs (48–50). This technique ablation may be expanded to intermediate stage HCC (51). However, CEUS has some detection limits for deep lesion, hypovascular HCC in a cirrhotic liver and lesions located in the subdiaphragrnatic regions. Therefore, we would like to recommend you choose fusion imaging guidance first.

## Conclusion

CEUS and fusion imaging are relevant to identify HCC with poor conspicuity. Therefore, operators can perform ablation therapy with more confidence. CEUS/fusion imaging guidance has improved the clinical effectiveness of ablation therapy in poorly conspicuous HCC patients. However, CEUS or fusion imaging is limited in some situations. For example, HCC is unclear on CEUS either because the tumor location is deeper than 10 cm, the CT/MRI and US images could not be finally well-matched, or HCC is hidden behind bone or lung/bowl air. To overcome such situations, understanding the characteristics of each imaging guidance technique is key to identifying and managing poor conspicuous HCCs. No hostile relationship for ablation guidance between CEUS and fusion imaging. Occasionally, we can choose the combined guidance CEUS with fusion imaging in the most difficult situations. At least, we have to refrain from performing ablation therapy for poor conspicuous HCC with a simplistic strategy. CEUS/fusion imaging guided ablation therapy can provide longer recurrence-free survival rates and lower local tumor progression rates. Therefore, CEUS and fusion imaging can support the development of so-called “precise ablation.”

## Author Contributions

All authors listed have made a substantial, direct, and intellectual contribution to the work and approved it for publication.

## Conflict of Interest

The authors declare that the research was conducted in the absence of any commercial or financial relationships that could be construed as a potential conflict of interest.

## Abbreviations

CEUS, contrast-enhanced ultrasound; CT, computed tomography; HCC, hepatocellular carcinoma; MPR, multiplanar reconstruction; MRI, magnetic resonance imaging; MWA, microwave ablation; RFA, radiofrequency ablation; US, ultrasound; 2D, two-dimensional; 3D, three-dimensional.
